# Transcriptomic and metabolomic analyses reveal the mechanism of uniconazole inducing hypocotyl dwarfing by suppressing BrbZIP39–*BrPAL4* module mediating lignin biosynthesis in flowering Chinese cabbage

**DOI:** 10.3389/fpls.2022.1014396

**Published:** 2022-12-14

**Authors:** Liang Zhang, Min Zhong, Lingqi Yue, Xirong Chai, Puyan Zhao, Yunyan Kang, Xian Yang

**Affiliations:** College of Horticulture, South China Agricultural University, Guangzhou, Guangdong, China

**Keywords:** *Brassica campestris* L. ssp. *chinensis* var. *utilis* Tsen et Lee, uniconazole, hypocotyl elongation, phenylpropanoid biosynthesis pathway, basic leucine zipper

## Abstract

Uniconazole, a triazole plant growth regulator, is widely used to regulate plant height and prevent the overgrowth of seedlings. However, the underlying molecular mechanism of uniconazole in inhibiting the hypocotyl elongation of seedlings is still largely unclear, and there has been little research on the integration of transcriptomic and metabolomic data to investigate the mechanisms of hypocotyl elonga-tion. Herein we observed that the hypocotyl elongation of flowering Chinese cabbage seedings was significantly inhibited by uniconazole. Interestingly, based on combined transcriptome and metabolome analyses, we found that the “phenylpropanoid biosynthesis” pathway was significantly affected by uniconazole. In this pathway, only one member of the portal enzyme gene family, named *BrPAL4*, was remarkably downregulated, which was related to lignin biosynthesis. Furthermore, the yeast one-hybrid and dual-luciferase assays showed that BrbZIP39 could directly bind to the promoter region of *BrPAL4* and activate its transcript. The virus-induced gene silencing system further demonstrated that *BrbZIP39* could positively regulate hypocotyl elongation and the lignin biosynthesis of hypocotyl. Our findings provide a novel insight into the molecular regulatory mechanism of uniconazole inhibiting hypocotyl elongation in flowering Chinese cabbage and confirm, for the first time, that uniconazole decreases lignin content through repressing the BrbZIP39–*BrPAL4* module-mediated phenylpropanoid biosynthesis, which leads to the hypocotyl dwarfing of flowering Chinese cabbage seedlings.

## 1 Introduction

The elongation of hypocotyl is a complex and orderly process, which is affected by external and internal factors (Li et al., 2020; Khalaki et al., 2021). Understanding the molecular mechanism of hypocotyl elongation can establish the foundation for strong seedling cultivation (Song et al., 2022). The intensive production of vegetable seedlings with porous connected potholes as containers and artificial mixing substrates instead of soil often results in leggy seedlings. The hypocotyl length or hypocotyl diameter of leggy seedlings significantly increases or decreases the ratio of plant height to hypocotyl diameter increase. In addition, leggy seedlings display lower chlorophyll contents and elongated and weak hypocotyls which greatly influence their subsequent field performance (Zhou et al., 2019). Therefore, the control of hypocotyl overgrowth is a crucial part of the seedling stage.

Plant hormones significantly regulate plant height. Reducing bioactive gibberellins (GAs) has been widely employed for controlling stem elongation in various plants (Yan et al., 2021)—for example, *AtGA2ox7* or *AtGA2ox8* overexpression leads to a dwarf phenotype in both tobacco (*Nicotiana tabacum*) and *Arabidopsis* (Schomburg et al., 2003). Overexpressing *PcGA2ox1* of transgenic *Solanum nigrum* and *Solanum melanocerasum* from a runner bean resulted in a dwarf phenotype (Dijkstra et al., 2008). The overexpressing *OsGA2ox5* of rice leads to a dwarf plant with a shorter stem (Shan et al., 2014). *BnGA2ox2* and *BnGA2ox6* overexpression in *Arabidopsis* inhibits hypocotyl and stem growth (Yan et al., 2017; Yan et al., 2021). Moreover, biosynthesis inhibitor brassinazole of brassinosteroid (BR) restrains tomato hypocotyl growth through inhibiting the canonical cell cycle in the longitudinal direction, which is related to downregulating the expression of cell cycle genes (Song et al., 2022). Melatonin is drop by knocking out the pivotal enzyme gene such as the serotonin N-acetyltransferase 2 and tryptophan decarboxylase that is involved in the melatonin biosynthesis, which result in the *DWARF4* expression downregulating and endogenous BR levels decreasing as well as a semi-dwarf phenotype of rice (Lee and Back, 2018). It is reported that mutants with a deficient auxin synthesis exhibit shorter hypocotyl length, whereas mutants with an excessive auxin synthesis in the light display a longer hypocotyl length (Cheng et al., 2006). These previous investigations reveal that the growth and development of hypocotyls essentially need an appropriate concentration of auxin, and auxin can successfully affect hypocotyl length by promoting IAA3 degradation, which in turn results in the transcription factor of ARF being released (Vernoux et al., 2011; Oh et al., 2014). It can be seen that previous investigations have mainly conducted to explore the molecular mechanism of endogenous growth hormones on seedling dwarfing, but few studies have been focused on probing the molecular regulatory mechanism of exogenous regulators on hypocotyl dwarfing.

The plant growth regulators of triazole, such as uniconazole and paclobutrazol, can shorten internodes and dwarf plants (Tesfahun and Menzir, 2018). Uniconazole is a triazole compound that has similar structural and biological effects to paclobutrazol, and it is four to 10 times more active than paclobutrazol in controlling plant height (Blanchard and Runkle, 2007; Carver et al., 2014). Moreover, it has a shorter degradation cycle and less application dosage, and it is more environmentally friendly (Tao et al., 1997). Previous reports showed that uniconazole is widely applied to control plant height and enhance plant lodging resistance (Ahmad et al., 2021a; Lv et al., 2022). To date, uniconazole treatment in the process of intensive vegetable seedling cultivation mostly focuses on the screening of the application concentration (Zuo et al., 2020), and there is little information about its molecular regulatory mechanism. The key pathway of uniconazole on plant dwarfing remains largely unknown.

Basic leucine zipper (bZIP), which is widely existing in plants, is a family of transcription factors. It is involved in the growth and development of many plants and in response processes to biotic and abiotic stresses (Hossain et al., 2010; Tak and Mhatre, 2013). CAbZIP1 is involved in plant development and function as a possible regulator in increased disease resistance and environment stress tolerance (Lee et al., 2006). Fukazawa et al. (2000) indicated that bZIP regulates the morphology of plants by controlling the endogenous contents of GAs. Van Leene et al. (2016) showed that bZIP29 is involved in the leaf and root development of *Arabidopsis*. ZmbZIP4 can enhance root development in maize (Ma et al., 2018). Moreover, Weiste et al. (2017) demonstrated that AtbZIP11 can efficiently inhibit the expression of auxin-transport-promoting gene family *PIN-FORMED*, resulting in root growth retardation. bZIP39, a member of the A subgroup of the bZIP family, is extensively involved in many development processes in plants (Dröge-Laser et al., 2018)—for example, bZIP39 is extensively characterized concerning its function of ABA-dependent maturation and germination of seed (Skubacz et al., 2016) and takes part in floral transition regulation of plant by directly influencing *FLC* transcription, which suppresses the florigen gene *FT* (Shu et al., 2016; Shu et al., 2018). Furthermore, bZIP39 can also directly control genes linked with abiotic stress responses (Finkelstein and Lynch, 2000). Nevertheless, the available investigations on the molecular regulation mechanism of hypocotyl dwarfing in crops under the impact of bZIP are limited.

Flowering Chinese cabbage (*Brassica campestris* L. ssp. *chinensis* var. *utilis* Tsen et Lee) belongs to the Brassica, which is a famous, annually produced cruciferous vegetable widely distributed in China (Wang et al., 2022; Yue et al., 2022). In recent years, the production scale of flowering Chinese cabbage has been expanding, and its cultivation mode has changed from conventional direct sowing to intensive seedling transplantation; however, during the process of intensive seedling transplantation, leggy seedlings easily occur due to the overgrowth of hypocotyl, which leads to the decline of seedling quality. Thus, how to control the excessive growth of hypocotyl is an urgent problem to be solved in intensive seedling transplantation of flowering Chinese cabbage. So far, there has been little research on the integration of transcriptomic and metabolomic data to investigate the mechanisms of hypocotyl elongation. The molecular regulatory mechanism of uniconazole involved in regulating the overgrowth of hypocotyl in flowering Chinese cabbage has not been explored. We aimed to identify which genes and molecular pathways are responsive to hypocotyl dwarfing of seedlings mediated by uniconazole. By transcriptomic and metabolomic analyses, yeast one-hybrid, dual-luciferase, and virus-induced gene silencing (VIGS) assay, we revealed that uniconazole could induce hypocotyl dwarfing through inhibiting lignin biosynthesis in flowering Chinese cabbage seedlings. Our findings provide a novel insight into the molecular regulatory mechanism of uniconazole involved in inhibiting hypocotyl elongation through repressing the BrbZIP39–*BrPAL4* module-mediated phenylpropanoid biosynthesis. These results might have important practical implications for improving commercial seedling quality and greatly assist in guaranteeing the yield and quality of vegetables.

## 2 Materials and methods

### 2.1 Materials and treatments

The cultivar ‘Sijiu-19’ of flowering Chinese cabbage was used in the present study. The seeds were sowed using substrate plugs. After sowing, the seedling plugs were placed in artificial climatic chambers (RXZ-500D, Ningbo Jiangnan Instrument Factory, China) with a light intensity of 3,000 lx, relative humidity of 85%, and day (16 h)/night (8 h) temperature of 28°C/20°C. Five uniconazole concentrations of 0 mg·L^-1^ (pure water, CK), 25 mg·L^-1^ (T1), 50 mg·L^-1^ (T2), 100 mg·L^-1^ (T3), and 200 mg·L^-1^ (T4) were applied in the experiment. The different concentrations of uniconazole were sprayed into each seedling plug at 48 h after sowing, respectively, and the application dosage of each concentration was 100 ml per replicate. All treatments were carried out with three biological replicates. The length and diameter of the hypocotyls were measured by a vernier caliper at 9 days after treatment.

### 2.2 Histological observation

The hypocotyls were collected and fixed in formaldehyde–acetic acid–ethanol fixative. The tissue section method was as described in Zhang et al. (2020). Observation was conducted using a light microscope after making the sections (10 μm).

### 2.3 Determination of lignin content

The lignin content of hypocotyls was determined using a plant lignin ultraviolet spectrophotometer kit (Solarbio, Beijing, China). Briefly, the hypocotyl samples were dried at 80°C and then ground to powder. Moreover, 10 mg of the samples was placed into test tubes, and then 1 ml acetyl bromide solution (25% v/v acetyl bromide in glacial acetic acid) and 40 µl perchloric acid were added, fully mixed, and heated for 40 min at 80°C with vortexing every 10 min. After cooling (room temperature), 1 ml of a solution of glacial acetic acid and sodium hydroxide was added to end the reaction. Finally, after centrifugation at 8,000 *g* for 10 min, 20 µl of supernatant was mixed with 1.98 ml glacial acetic acid, and 1 ml liquid was added to a quartz cuvette to determine the absorbance using an ultraviolet spectrophotometer (Shimadzu Corp., Kyoto, Japan) at 280 nm. The results were expressed on dry weight basis as mg cm^-1^.

### 2.4 Transcriptome and metabolome analyses

The transcriptome and metabolome analyses of the hypocotyls in flowering Chinese cabbage treated with uniconazole were performed according to our previous report (Yue et al., 2022) based on the MetWare database (Metware Biotechnology Co., Ltd., Wuhan, China). The value of variable importance in the project (VIP) was calculated by orthogonal partial least square discriminant analysis. Differentially accumulated metabolites (DAMs) between the two groups were determined by VIP ≥1 and absolute log_2_(fold change, FC) ≥1. The differential expression analysis was consistent with our previous report (Yue et al., 2022). Differentially expressed genes (DEGs) between groups were determined by *P* ≤0.05 and absolute log_2_(FC) ≥1. The joint analysis was carried out to analyze the biological changes of the hypocotyls in flowering Chinese cabbage treated with uniconazole. DEGs and DAMs were mapped to the Kyoto Encyclopedia of Genes and Genomes (KEGG, https://www.kegg.jp/) pathway, and enrichment analysis was performed.

### 2.5 Yeast one-hybrid assay

The *BrbZIP39* sequence was ligated into the pGADT7 vector using the *Bam*Hι and *Eco*RI restriction sites. The *BrPAL4* promoter (1,899 bp upstream of the predicted translation start site) fragment was cloned into the pAbAi vector using the *Sac*I and *Xho*I restriction sites. They were then transferred into the cell of yeast strain Y1H Gold (TransGen, Beijing, China) through Yeastmaker™ Yeast Transformation System 2. Strains introduced with plasmids of pGADT7 and/or p53 served as negative and positive controls, respectively. The transformants were grown on SD/-Leu media and incubated at 30°C for 5 days.

### 2.6 Dual-luciferase reporter assay

Full-length ORFs of *BrbZIP39* were inserted into pGreenll 62-SK to generate the effector. In addition, the promoter fragment of *BrPAL4* was fused into pGreenll 0800-luciferase reporter (LUC) to obtain the reporter. The effector and reporter vectors were co-transformed into tobacco (*Nicotiana benthamiana*) leaves.

### 2.7 Virus-induced gene silencing assay in flowering Chinese cabbage

The vectors of pTRV1 and pTRV2 based on the tobacco rattle virus (TRV) were employed to silence the *BrbZIP39*. A 300-bp fragment of *BrbZIP39* was amplified and cloned into pTRV2 to generate pTRV2–*BrbZIP39*. Then, pTRV1, pTRV2, and pTRV2–*BrbZIP39* were transformed into GV3101, respectively. The GV3101 strains with pTRV2–*BrbZIP39* and pTRV1 were syringe-infiltrated into flowering Chinese cabbage which was designated as pTRV : *BrbZIP39*. A mixture of pTRV1 and pTRV2 empty was used as control (pTRV:00).

### 2.8 Quantitative real-time PCR

Total RNA was extracted from the samples using a plant RNA extraction kit (Magen, Guangzhou, China) according to the manufacturer’s instructions. qRT-PCR was performed using the Q711-ChamQTM Universal SYBR^®^ qPCR Master Mix (Vazyme, Nanjing, China) with Bio-Rad CFX96 Real-Time PCR System. The qRT-PCR reaction conditions were described as follows: 30 s at 95°C, 40 cycles of 10 s at 95°C, and 30 s at 60°C. The transcript abundance of 12 randomly selected DEGs was calculated according to the 2^−△△Ct^ method. *BrGAPDH* was used as the internal control. All primers used herein are listed in **Supplementary Table S3**.

### 2.9 Statistical analysis

All data were derived from three biological replicates and expressed as mean ± SE. R v4.1.1 and IBM SPSS Statistics 24 (SPSS Inc., Chicago, IL, USA) were used for statistical analysis. Tukey’s test (*P* < 0.05) was carried out to evaluate the treatment effects. All figures were generated using R v4.1.1 and Origin 2021 (OriginLab Corp., Northampton, MA, USA).

## 3 Results

### 3.1 The effects of uniconazole on hypocotyl elongation of seedlings

**Figure 1:**
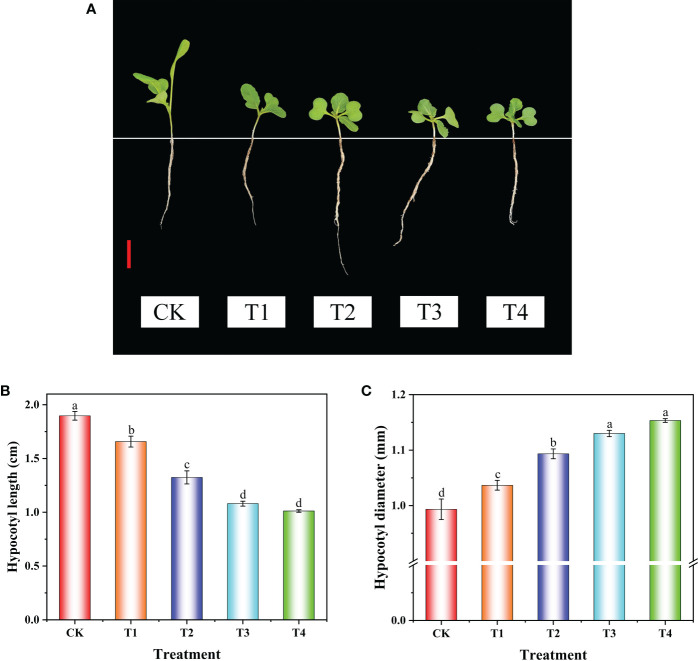
The phenotype and hypocotyl growth of flowering Chinese cabbage seedlings under uniconazole treatment. The phenotype **(A)**, length **(B)**, and diameter **(C)** of hypocotyl at 9 days after uniconazole treatment in flowering Chinese cabbage seedlings. CK, 0 mg·L^-1^ uniconazole (pure water); T1, 25 mg·L^-1^ uniconazole; T2, 50 mg·L^-1^ uniconazole; T3, 100 mg·L^-1^ uniconazole; T4, 200 mg·L^-1^ uniconazole. The vertical bars show the standard error of the mean (*n* = 10). Red bar, 2 cm. Letters above columns mean significant difference at *P <*0.05.

Uniconazole is widely applied to control plant height and enhance plant lodging resistance (Ahmad et al., 2021a; Lv et al., 2022). In this study, uniconazole treatment remarkably inhibited hypocotyl elongation and increased the hypocotyl diameter of flowering Chinese cabbage seedlings (**Figure 1A**). Compared with CK, the hypocotyl length of T1, T2, T3, and T4 treatments significantly decreased by 12.65%, 30.18%, 43.08%, and 46.67%, and the hypocotyl diameter of T1, T2, T3, and T4 treatments significantly increased by 4.36%, 10.07%, 13.76%, and 16.11%, respectively. Additionally, there was no significant difference in hypocotyl length and diameter between the T3 and T4 treatments (**Figures 1B, C**). These results showed that, with the increase of uniconazole concentration in the range of 0–100 mg·L^−1^, the hypocotyl length became shorter obviously, but there was no significant difference at the level of 100–200 mg·L^−1^. Based on the above-mentioned results, the CK and T3 (100 mg·L^−1^ uniconazole) treatments were selected for histological observation, metabolome detection, and transcriptome sequencing.

### 3.2 Histological observation of hypocotyl

**Figure 2:**
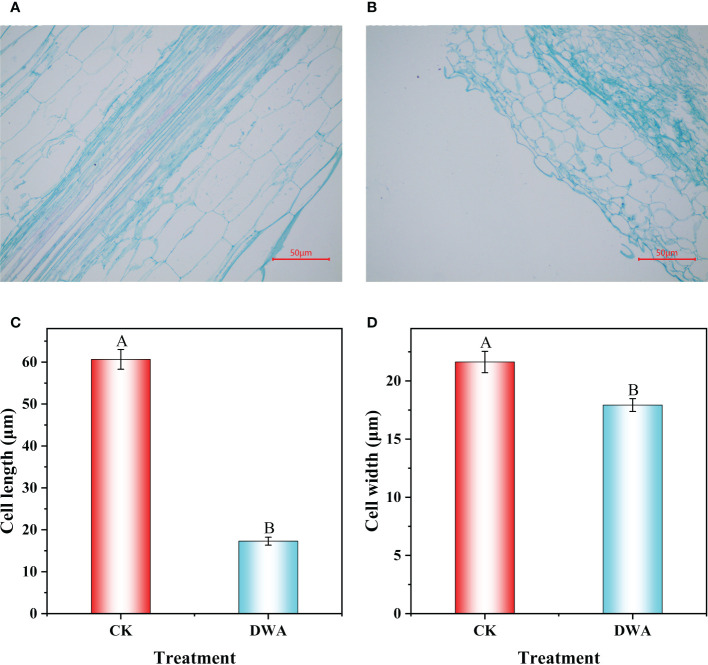
Longitudinal section and cell size of hypocotyl under uniconazole in flowering Chinese cabbage seedlings. **(A)** Parenchyma cells of CK. **(B)** Parenchyma cells of DWA. **(C)** Cell length. **(D)** Cell width. CK, 0 mg L^-1^ uniconazole (pure water); DWA, 100 mg L^-1^ uniconazole. The vertical bars show the standard error of the mean (*n* = 10). Letters above columns mean significant difference at *P <*0.01.

To further explore the cause of hypocotyl dwarfing induced by uniconazole, the hypocotyl was dissected at 9 days after treatment. We observed that the shape of the parenchyma cells changed significantly after uniconazole treatment (**Figures 2A, B**). The average cell width of hypocotyls was 17.93 and 21.63 μm, respectively, in uniconazole treatment and CK, which was about 1.2-folds wider than in uniconazole treatment (**Figure 2C**). The average cell length of hypocotyls was 60.65 μm in CK, while that in uniconazole treatment was only 17.29 μm (**Figure 2D**). That was to say, the cell morphology of the hypocotyl has changed dramatically after uniconazole treatment, which implied that the hypocotyl dwarfing of flowering Chinese cabbage seedlings may be related to the cell size.

### 3.3 Metabolome analysis

**Figure 3:**
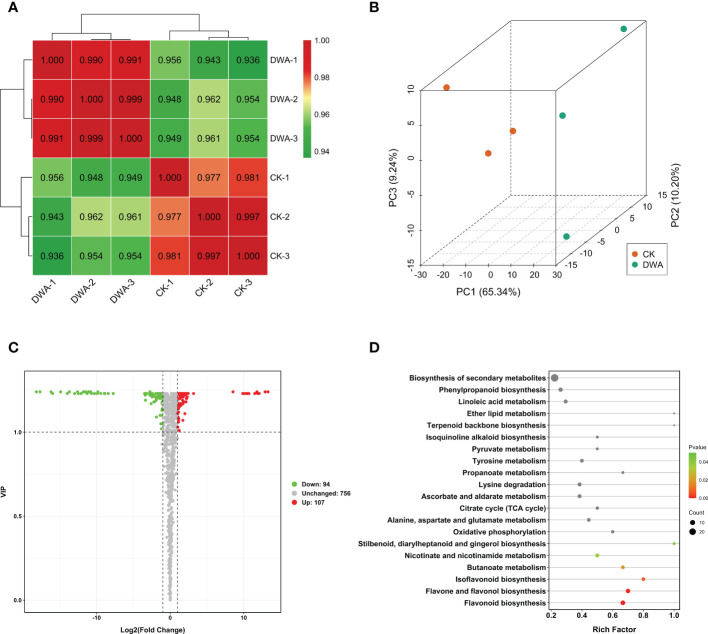
Metabolome analysis of flowering Chinese cabbage under uniconazole treatment. **(A)** Correlation profile between the samples. **(B)** Principal component analysis of metabolome samples. **(C)** Volcano map of DAMs. **(D)** Kyoto Encyclopedia of Genes and Genomes pathway enrichment of DAMs. DAMs, differentially accumulated metabolites; CK, 0 mg·L^-1^ uniconazole (pure water); DWA, 100 mg·L^-1^ uniconazole.

To profile the metabolic changes of hypocotyl dwarfing induced by uniconazole, we performed non-targeted metabolome detection using a UPLC-ESI-MS/MS system. We identified and annotated 467 and 479 metabolites in the tested samples. The quality of the metabolome data was good, showing high intra-group correlations (**Figure 3A**) and a clear separation trend in the principal component analysis (**Figure 3B**).

The results revealed that the metabolites in hypocotyls under uniconazole treatment were remarkably changed. The 201 DAMs including 94 downregulated and 107 upregulated metabolites were filtered out through setting a threshold value of absolute log_2_(FC) ≥1 and VIP ≥1 (**Figure 3C**). The KEGG enrichment analysis indicated that three flavonoid-related pathways including “flavonoid biosynthesis”, “flavone and flavonol biosynthesis”, and “isoflavonoid biosynthesis” were significantly overrepresented in DAMs (**Figure 3D**). Among the enriched DAMs, eight were associated with “flavonoid biosynthesis” pathway, seven were associated with “flavone and flavonol biosynthesis” pathway, four were associated with the “isoflavone biosynthesis” pathway, and six were associated with “phenylpropanoid biosynthesis” pathway (**Supplementary Figure S1**). Amid the top 10 DAMs, the quantities of flavonoids were all downregulated except qercetin-3-O-xyloside, and nine of the top 10 downregulated DAMs were flavonoids (**Supplementary Figure S2**). Considering that flavonoids are secondary metabolites derived from the pathway of phenylpropanoid, we speculated that the phenylpropanoid pathway may play a critical role in the hypocotyl dwarfing of flowering Chinese cabbage seedlings under uniconazole treatment.

### 3.4 Transcriptome analysis

The transcriptomes of the seedling hypocotyls from the CK and uniconazole treatment groups were applied by Illumina sequencing. A total of 41.48 Gb clean data was generated from the six samples with Q30 values being all higher than 93% (shown in **Supplementary Table S1**). The clean reads data were compared with the flowering Chinese cabbage genome (unpublished) by hisat2 v2.1.0; a total of 42,491 unigenes were obtained. Based on transcriptome annotation and functional classification, 97.53% (41,441) unigenes were assigned putative functional annotation (**Supplementary Figure S3**). Gene Ontology analysis showed that “transcription, DNA-templated” and “regulation of transcription, DNA-templated” containing 3,232 (11.48%) and 1,881 (6.68%) unigenes, respectively, were the two largest sub-categories in the biological process. The “nucleus” and “integral component of membrane”, which accounted for 6,771 (24.06%) and 6,611 (23.49%) unigenes, respectively, were the two largest sub-categories in the cellular component. “ATP binding” and “metal ion binding” contained 3,698 (13.14%) and 3,073 (10.92%) unigenes, respectively, and were the two sub-categories in the molecular function cluster (**Supplementary Figure S4**).

**Figure 4:**
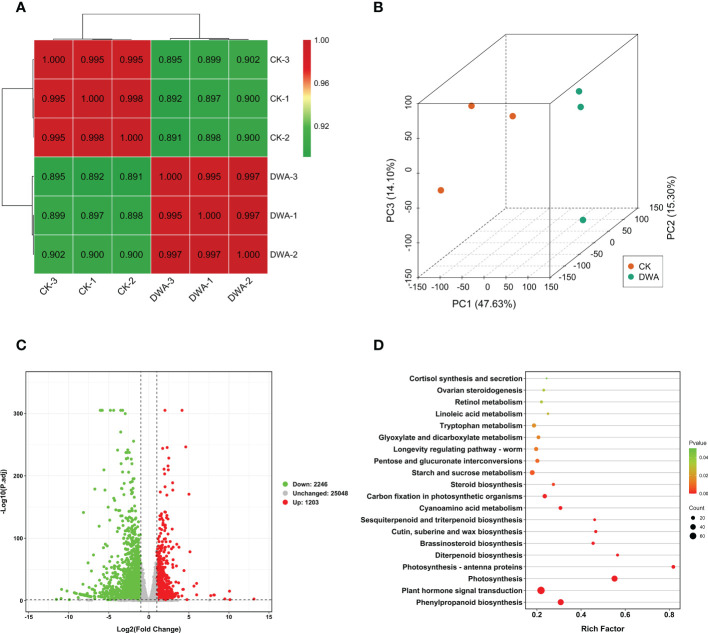
Transcriptome analysis of flowering Chinese cabbage under uniconazole treatment. **(A)** Correlation heat map between the samples (based on fragments per kilobase of transcript per million fragments mapped). **(B)** Principal component analysis of transcriptome samples. **(C)** Volcano map of DEGs. **(D)** Kyoto Encyclopedia of Genes and Genomes pathway enrichment of DEGs. DEGs, differentially expressed genes; CK, 0 mg·L^-1^ uniconazole (pure water); DWA, 100 mg·L^-1^ uniconazole.

The unigene expression levels were described as fragments per kilobase of transcript per million fragments mapped values. The calculation of the correlations between every two samples demonstrated high intra-group correlations (**Figure 4A**), and the principal component analysis indicated a clear separation trend (**Figure 4B**). The 3,449 DEGs including 2,246 downregulated and 1,203 upregulated unigenes were filtered out by setting a threshold of *P*-adj <0.05 and absolute log_2_ (FC) ≥1 (**Figure 4C**). The KEGG enrichment analysis revealed that two pathways—”plant hormone signal transduction” and “phenylpropanoid biosynthesis”—were significantly overrepresented in DEGs (**Figure 4D**).

### 3.5 Conjoint analysis of metabolome and transcriptome

**Figure 5:**
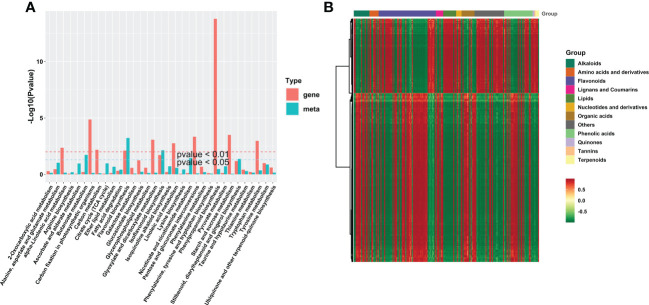
Conjoint analysis transcriptome and metabolome. **(A)** Kyoto Encyclopedia of Genes and Genomes pathway enrichment of DEGs and DAMs. **(B)** Clustered heat map of DEGs and DAMs. DAMs, differentially accumulated metabolites; DEGs, differentially expressed genes.

To confirm the relationship between metabolites and genes, we conducted a joint analysis of metabolome and transcriptome. The KEGG pathway enrichment analysis showed 33 co-enriched KEGG pathways of DEGs and DAMs (**Figure 5A**). Among them, “phenylpropanoid biosynthesis” pathway is the most significant enrichment pathway; “carbon fixation in photosynthetic organisms” pathway, “flavonoid biosynthesis” pathway, “pentose and glucuronate interconversions” pathway, “tryptophan metabolism” pathway, and “starch and sucrose metabolism” pathway are also significantly enriched. The clustered heat map (**Figure 5B**) revealed that the DAMs associated with DEGs were classified into several categories, with flavonoids as the largest category, which indicated that “phenylpropanoid biosynthesis” pathway plays a crucial role in hypocotyl dwarfing.

### 3.6 Effects of uniconazole on lignin content and *BrPALs* and *BrbZIPs* expression in hypocotyls

**Figure 6:**
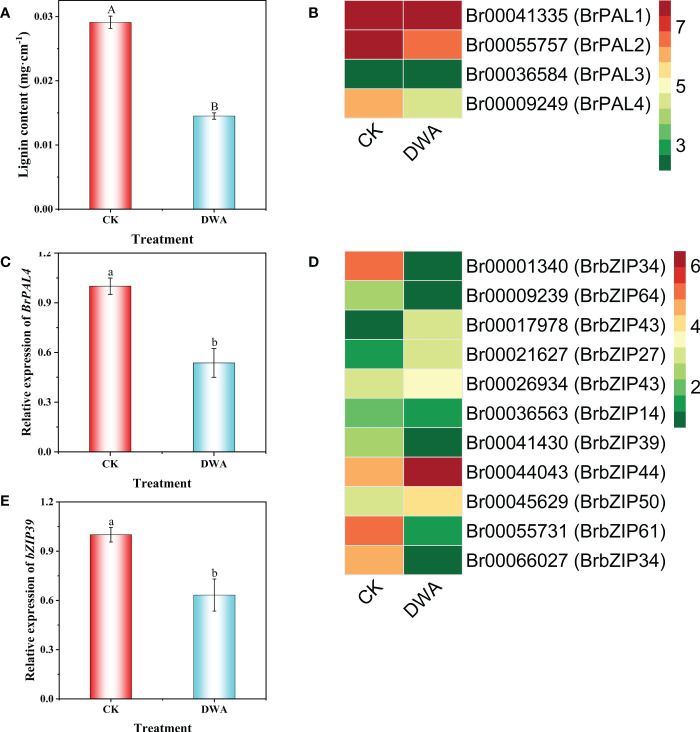
Effects of uniconazole on lignin content, *BrPALs* and *BrbZIPs* expression of hypocotyls in flowering Chinese cabbage. Lignin content **(A)**, fragments per kilobase of transcript per million fragments mapped (FPKM) of *BrPALs* in DEGs **(B)**, relative expression of *BrPAL4* **(C)**, FPKM of *BrbZIPs* in DEGs **(D)**, and relative expression of *BrbZIP39* **(E)** in hypocotyl of flowering Chinese cabbage seedlings under CK and DWA. The vertical bars show the standard error of the mean (*n* = 3). CK, 0 mg·L^-1^ uniconazole (pure water); DWA, 100 mg·L^-1^ uniconazole. Letters above columns mean significant difference at *P <*0.05.

Based on the omics analysis, we speculated that hypocotyl dwarfing of flowering Chinese cabbage seedlings caused by uniconazole was associated with lignin biosynthesis. To confirm the hypothesis, we determined the lignin content of hypocotyl, and the results demonstrated that the lignin content of the hypocotyl in uniconazole treatment was remarkably decreased by 50.15% compared with CK (**Figure 6A**). Surprisingly, we found only one member (*Br00009249*) in *BrPALs* among DEGs, which was significantly downregulated after uniconazole treatment (**Figures 6B, C**). We applied the protein sequence of *Br00009249* as the query to BLASTP against the *Arabidopsis* genome database; it was named *BrPAL4*. Moreover, uniconazole treatment also downregulated most of the *BrbZIPs* family genes, including *BrbZIP39* (**Figures 6D, E**). These findings showed that uniconazole inhibiting hypocotyl elongation was associated with lignin content, *BrPAL4*, and *BrbZIP39* expression.

### 3.7 Activation of the *BrPAL4* promoter by bZIP39

To identify the regulatory relationship between BrbZIP39 and *BrPAL4*, we further analyzed the cis-acting elements of *BrPAL4*. The promoter region of *BrPAL4* (upstream 2,000 bp) contained nine ABRE and four G-box that could be bound by bZIP39 (**Supplementary Table S2**). Therefore, we inferred that uniconazole probably regulated *BrPAL4* through mediating BrbZIP39 expression, which might lead to hypocotyl dwarfing of flowering Chinese cabbage seedlings.

**Figure 7:**
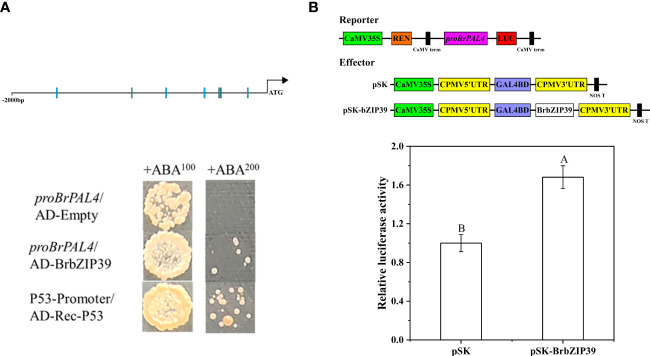
BrbZIP39 could activate *BrPAL4* expression. **(A)** Yeast one-hybrid analysis showed that BrbZIP39 could bind to the promoter of *BrPAL4*. **(B)** Dual-luciferase assay indicated that BrbZIP39 was the activator of *BrPAL4*.

To verify whether BrbZIP39 has a regulatory effect on *BrPAL4*, we performed Y1H and LUC experiments. The results displayed that the Y1H Gold yeast transformed with pGADT7-BrbZIP39 in deficient media SD/-Leu grew normally, the negative control yeast transformed with pGADT7 did not grow, and the positive control yeast transformed with p53 grew normally (**Figure 7A**). Additionally, compared with the control that was cotransfected with the empty pSK vector, the relative luciferase activity of pSK-BrbZIP39 increased significantly (**Figure 7B**). The above-mentioned findings demonstrated that BrbZIP39 could directly bind to the promoter region of *BrPAL4* and activate its transcript, indicating that BrbZIP39 is the transcriptional activator of *BrPAL4*.

### 3.8 Silencing *BrbZIP39* caused hypocotyl dwarfing in flowering Chinese cabbage

**Figure 8:**
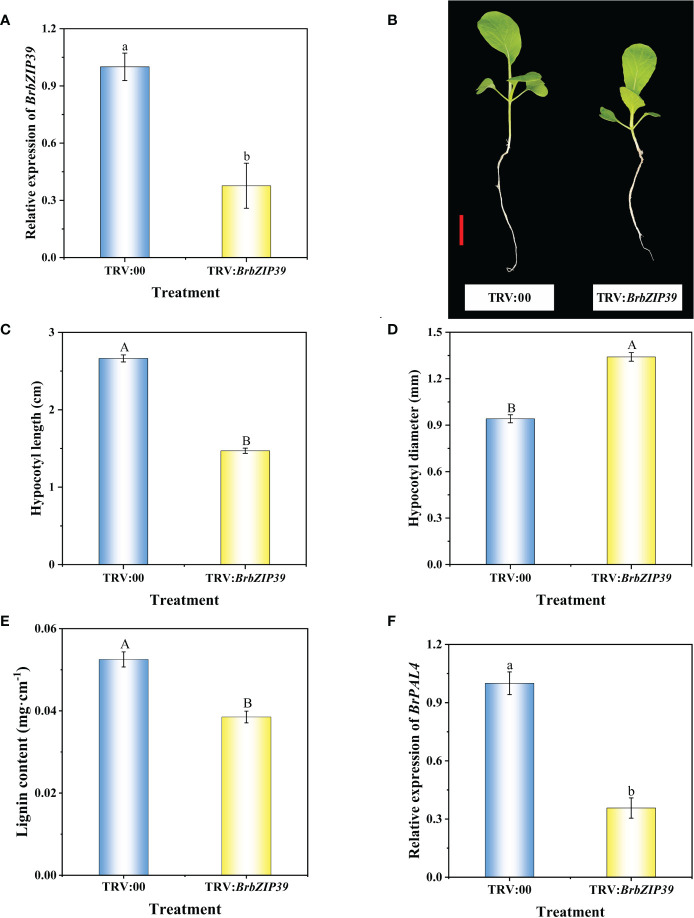
Silencing of *BrbZIP39* in flowering Chinese cabbage by virus-induced gene silencing. The phenotype **(A)**, relative expression of *BrbZIP39* **(B)**, hypocotyl length **(C)**, hypocotyl diameter **(D)**, relative expression of *BrPAL4* **(E)**, and lignin content **(F)** of TRV:00 and TRV : *BrbZIP39* plants in flowering Chinese cabbage. The vertical bars show the standard error of the mean (*n* = 6). Red bar, 2 cm. Letters above columns mean significant difference at *P* < 0.05.

To unravel whether *BrbZIP39* is required for hypocotyl dwarfing, the VIGS system was employed to silence *BrbZIP39* in flowering Chinese cabbage. The results displayed that *BrbZIP39* expression in the hypocotyl was significantly decreased after silencing *BrbZIP39*, and the *BrbZIP39* transcript abundance in TRV : *BrbZIP39* plants was remarkably reduced by 62.33% compared with that of TRV:00 (**Figure 8A**). Meanwhile, compared with TRV:00, the hypocotyl length of TRV : *BrbZIP39* was significantly decreased by 44.78% (**Figures 8B, C**), and the hypocotyl diameter of TRV : *BrbZIP39* was significantly increased by 42.42% (**Figures 8B, D**). Furthermore, the lignin content and the expression of *BrPAL4* in the hypocotyl were significantly decreased in TRV : *BrbZIP39* plants (**Figures 8E, F**). These results further confirmed that uniconazole decreased the lignin content through repressing BrbZIP39–*BrPAL4* module-mediated phenylpropanoid biosynthesis, which resulted in the hypocotyl dwarfing of flowering Chinese cabbage seedlings.

### 3.9 qRT-PCR validation

To verify the results obtained in the RNA-seq experiments, the expressions of 12 DEGs were analyzed using qRT-PCR. The transcriptome sequencing and bioinformatics analyses showed that *Br00009249*, *Br00020065*, *Br00036384*, *Br00043806*, *Br00055757*, *Br00058057*, and *Br00062465* were downregulated, while *Br00030167*, *Br00052005*, *Br00066177*, *Br00066395*, and *Br00067601* were upregulated under uniconazole treatment. The qRT-PCR results revealed that the expression patterns of these representative genes were in line with the RNA-seq data (**Supplementary Figure S5A**). To evaluate RNA-seq data, linear regression analysis was performed, and he results showed that the data from qRT-PCR and RNA-seq of the 12 DEGs were obviously correlated (*R* ^2^ = 0.66008, *P* < 0.001, **Supplementary Figure S5B**), implying the reliability of the RNA-Seq data.

## 4 Discussion

The hypocotyl length, closely associated with the biomass and the yield of plants, is an important component of plant architecture (Zhang et al., 2019). Factory-raised seedlings, at present, are widely applied in vegetable production; however, the conditions of growth in factories—including low light, high humidity, and high temperature—result in the excessive growth of hypocotyls during seedling cultivation and obviously affect the survival rate of vegetable seedlings (Gomez and Mitchell, 2014; Song et al., 2022). Uniconazole, a plant growth regulator, can effectively decrease plant height, increase root–shoot ratio and stem diameter, and enhance lodging resistance (Currey et al., 2016; Ahmad et al., 2020; Zuo et al., 2020). In our present study, uniconazole treatment significantly decreased plant height and hypocotyl length but enhanced hypocotyl diameter, and the level of increase or decrease was correlated with the applied concentration in a certain range (0–100 mg·L^-1^) in flowering Chinese cabbage (**Figures 1A–C**). These results suggested that uniconazole could effectively control hypocotyl overgrowth and be used in the cultivation of strong seedlings of flowering Chinese cabbage. In addition, previous studies indicated that BR-deficient or BR-insensitive mutants displayed a dwarf phenotype of the plant due to shortened cell length (Gupta et al., 2021). In this study, the histological observation showed that cell length was obviously shortened under uniconazole treatment (**Figure 2C**), which was similar to previous studies (Kato et al., 2011; Lv et al., 2022). This finding indicated that shortened cells might be a response to the decrease of hypocotyl length, and the enlargement of parenchyma cells might be a response to the increase of hypocotyl diameter (**Supplementary Figure S6**).

Although uniconazole has a wide application in crop production, the molecular mechanism about inhibiting hypocotyl elongation mediated by uniconazole remains largely unknown. To investigate the molecular regulatory mechanism of uniconazole involved in inhibiting hypocotyl elongation, we conducted transcriptomic and metabolomic analyses. The dataset provided a useful resource for the clarification of the key dwarfing pathways not yet deciphered in seedling hypocotyl under uniconazole treatment in flowering Chinese cabbage. Our investigation indicated that a total of 3,499 DEGs and 201 DAMs were affected by uniconazole (**Figures 4C**, **6C**), and the KEGG pathway analysis demonstrated that most DEGs were remarkably enriched in the pathway of “plant hormone signal transduction” (**Figure 6D**). These findings also explain why the previous research on dwarf phenotype mainly focused on endogenous hormone changes in plants (Liu et al., 2015; Ahmad et al., 2021b; Zhou et al., 2021). Interestingly, in this study, “phenylpropanoid biosynthesis” pathway, which is involved in hypocotyl dwarfing of flowering Chinese cabbage, was proposed for the first time by combined metabolome and transcriptome analyses. The joint metabolome and transcriptome analyses revealed that the DEGs were significantly enriched in “phenylpropanoid biosynthesis” pathway, while the DAMs were significantly enriched in “flavonoid biosynthesis” pathway. Considering that flavonoids are secondary metabolites derived from the pathway “phenylpropanoid biosynthesis”, we thus speculated that “phenylpropanoid biosynthesis” pathway plays a vital role in hypocotyl dwarfing induced by uniconazole in flowering Chinese cabbage seedlings.

Previous studies have also pointed out that there is a close relationship between plant dwarfing and lignin, which is an important phenylpropanoid. Li et al. (2010) have indicated that the dwarf phenotype results instead from disruptions in lignin biosynthesis. The dwarf mutant with *cinnamoyl-CoA reductase* deficiency has significantly less lignin, while the cellulose and hemicellulose levels are unchanged, implying that plant dwarfing is related to lignin (Goujon et al., 2003). The mutation of *UGT72B1* results in ectopic lignification and lignin biosynthesis, which is responsible for the glycosylation of monolignols that leads to growth suppression (Lin et al., 2016). The *lac4*, *lac11*, and *lac17* triple mutation in *Arabidopsis* lacks lignification in the vascular tissues, which represses plant growth (Zhao et al., 2013). The irregular xylem4 mutants accounted for about 50% less lignin content than the wild type, have collapsed xylem, and are seriously dwarfed (Jones et al., 2001). The overexpression of R2R3-MYB transcription factor ZmMYB31 also shows a dwarf phenotype, while the lignin content is significantly decreased in *Arabidopsis thaliana* (Fornalé et al., 2010). Our results displayed that the lignin level in the hypocotyl of the dwarf phenotype was significantly decreased in flowering Chinese cabbage seedlings (**Figures 3A, B**). This finding further confirmed that the inhibition of hypocotyl elongation caused by uniconazole was related to the phenylpropanoid biosynthesis pathway. However, how does uniconazole cause hypocotyl dwarfing by inhibiting the formation of lignin? The knowledge in this area is still limited.

Phenylalanine ammonia-lyase (PAL) is a portal enzyme in the pathway of phenylpropanoid biosynthesis (Barros et al., 2016). *PALs* family contains multiple family members; however, it has not identified which members are the pivotal regulators in inducing hypocotyl dwarfing of flowering Chinese cabbage seedlings mediated by uniconazole. Different *PAL* gene members have different functions in plants—for example, *PAL1* and *PAL2* have functionally participated in flavonoid synthesis; *PAL4* may be a good candidate for playing an important role in lignin synthesis (Olsen et al., 2008). Previous reports found that *pal1 pal2* mutant reduces lignin content, with deficiency in anthocyanin and tannin biosynthesis (Rohde et al., 2004; Huang et al., 2010). Furthermore, Rohde et al. (2004) also noted that mutations in each of *PAL1* and *PAL2* alone do not exhibit a noticeable dwarfing phenotype. In this study, we found that *BrPAL4* was downregulated in the hypocotyl of the dwarf phenotype caused by uniconazole, accompanied by a decrease of lignin content (**Figure 6**). Our results further demonstrated that “phenylpropanoid biosynthesis” pathway played an important role in the hypocotyl dwarfing of flowering Chinese cabbage seedlings, while the reduction of lignin content was a reason for the hypocotyl dwarfing induced by uniconazole.

bZIP transcription factors are widely existing in eukaryotes and involve in the growth and development of many plants (Fukazawa et al., 2000). HY5, a variety of the bZIP transcription factor family, plays a vital role in hypocotyl elongation in plants (Oyama et al., 1997). Li et al. (2011) showed that HY5 can mediate AtERF11 to inhibit ethylene biosynthesis in *Arabidopsis*, thereby affecting seedling growth and development. It has been reported that *bZIP30* overexpression induces a dwarf phenotype and affects reproductive development in *Arabidopsis*, while loss-of-function mutants for *bZIP30* and its closest homolog have more floral buds and longer fruits (Lozano-Sotomayor et al., 2016). Overexpression of *bZIP53* transgenic *Arabidopsis* also shows a dwarf phenotype associated with reduced seed production and an elevated gene expression of seed maturation genes (Alonso et al., 2009; Garg et al., 2019). Taken together, the bZIP transcription factors play a vital role in the growth and development of plants. Our results also demonstrated that uniconazole could downregulate the expression of *BrbZIP39*, indicating that *BrbZIP39* is involved in the regulation of hypocotyl dwarfing in flowering Chinese cabbage. However, to date, the previous investigation focused mainly on bZIP transcription factors and the effects of photomorphogenesis and reproductive development in plants; there were few reports on the effects of “phenylpropanoid biosynthesis” pathway which is a key pathway between the primary and secondary metabolic pathways. Moreover, it is still unclear whether bZIP transcription factors can regulate hypocotyl dwarfing by mediating *PAL* expression and lignin biosynthesis. To determine whether BrbZIP39 could transcriptionally activate *BrPAL4*, we performed the yeast one-hybrid and dual-luciferase assays. Our results exhibited that BrbZIP39 could directly bind to the promoter region of *BrPAL4* and activate its transcription (**Figure 7**). To further verify whether BrbZIP39 could dwarf the hypocotyl of flowering Chinese cabbage seedlings by reducing the content of lignin in the hypocotyl, we silenced *BrbZIP39* using the VIGS system. We found that positive plants had shorter hypocotyls and that there was less lignin content in the hypocotyl of flowering Chinese cabbage seedlings (**Figure 8**). These results confirmed that BrbZIP39 was related to the dwarfing of hypocotyl. Hence, we further proved that the inhibition of BrbZIP39–*BrPAL4* decreased the content of lignin in the hypocotyl, which was a reason for hypocotyl dwarfing mediated by uniconazole. Taken together, our results propose that uniconazole can decrease lignin content *via* repressing BrbZIP39–*BrPAL4* module-mediated phenylpropanoid biosynthesis, which leads to hypocotyl dwarfing of flowering Chinese cabbage seedlings. Our present findings provide a new insight into the molecular regulatory mechanism and enrich our understanding of inhibiting hypocotyl elongation in flowering Chinese cabbage.

## Data availability statement

The datasets presented in this study can be found in online repositories. The names of the repository/repositories and accession number(s) can be found below: https://www.ncbi.nlm.nih.gov/, PRJNA855288.

## Author contributions

XY designed the research. LZ, MZ, and LY performed the experiments and wrote the paper. XC and PZ analyzed the data. YK and MZ reviewed the manuscript. All authors contributed to the article and approved the submitted version.

## References

[B1] Ahmad I. Ahmad S. Yang X. N. Meng X. P. Yang B. P. Liu T.. (2021a). Effect of uniconazole and nitrogen level on lodging resistance and yield potential of maize under medium and high plant density. Plant Biol. 23, 485–496. doi: 10.1111/plb.13235 33423379

[B2] Ahmad I. Kamran M. Meng X. P. Ali S. Ahmad S. Gao Z. Q.. (2021b). Hormonal changes with uniconazole trigger canopy apparent photosynthesis and grain filling in wheat crop in a semi-arid climate. Protoplasma 258, 139–150. doi: 10.1007/s00709-020-01559-0 32968872

[B3] Ahmad I. Meng X. P. Kamran M. Ali S. Ahmad S. Liu T. N.. (2020). Effects of uniconazole with or without micronutrient on the lignin biosynthesis, lodging resistance, and winter wheat production in semiarid regions. J. Integr. Agricult. 19, 62–77. doi: 10.1016/S2095-3119(19)62632-8

[B4] Alonso R. Onate-Sánchez L. Weltmeier F. Ehlert A. Diaz I. Dietrich K.. (2009). A pivotal role of the basic leucine zipper transcription factor bZIP53 in the regulation of arabidopsis seed maturation gene expression based on heterodimerization and protein complex formation. Plant Cell. 21, 1747–1761. doi: 10.1105/tpc.108.062968 19531597 PMC2714925

[B5] Barros J. Serrani-Yarce J. C. Chen F. Baxter D. Venables B. J. Dixon R. A. (2016). Role of bifunctional ammonia-lyase in grass cell wall biosynthesis. Nat. Plants 2, 16050. doi: 10.1038/nplants.2016.50 27255834

[B6] Blanchard M. G. Runkle E. S. (2007). Dipping bedding plant liners in paclobutrazol or uniconazole inhibits subsequent stem extension. Hort. Technol. Hort. 17, 178–182. doi: 10.21273/HORTTECH.17.2.178

[B7] Carver S. T. Arnold M. A. Byrne D. H. Armitage A. R. Daniel Lineberger R. King A. R. (2014). Growth and flowering responses of sea marigold to daminozide, paclobutrazol, or uniconazole applied as drenches or sprays. J. Plant Growth Regulation 33, 626–631. doi: 10.1007/s00344-014-9411-7

[B8] Cheng Y. F. Dai X. H. Zhao Y. D. (2006). Auxin biosynthesis by the YUCCA flavin monooxygenases controls the formation of floral organs and vascular tissues in arabidopsis. Genes Dev. 20, 1790–1799. doi: 10.1101/gad.1415106 16818609 PMC1522075

[B9] Currey C. J. Flax N. J. Walters K. J. (2016). Foliar sprays of flurprimidol, paclobutrazol, and uniconazole suppress height of seed-propagated new guinea impatiens. Hort. Technol. Hort. 26, 20–25. doi: 10.21273/HORTTECH.26.1.20

[B10] Dijkstra C. Adams E. Bhattacharya A. Page A. F. Anthony P. Kourmpetli S.. (2008). Over-expression of a *gibberellin 2-oxidase* gene from *Phaseolus coccineus* l. enhances gibberellin inactivation and induces dwarfism in *Solanum* species. Plant Cell Rep. 27, 463–470. doi: 10.1007/s00299-007-0471-z 17999064

[B11] Dröge-Laser W. Snoek B. L. Snel B. Weiste C. (2018). The arabidopsis bZIP transcription factor family — an update. Curr. Opin. Plant Biol. 45, 36–49. doi: 10.1016/j.pbi.2018.05.001 29860175

[B12] Finkelstein R. R. Lynch T. J. (2000). The arabidopsis abscisic acid response gene *ABI5* encodes a basic leucine zipper transcription factor. Plant Cell. 12, 599–609. doi: 10.1105/tpc.12.4.599 10760247 PMC139856

[B13] Fornalé S. Shi X. H. Chai C. L. Encina A. Irar S. Capellades M.. (2010). ZmMYB31 directly represses maize lignin genes and redirects the phenylpropanoid metabolic flux. Plant J. 64, 633–644. doi: 10.1111/j.1365-313X.2010.04363.x 21070416

[B14] Fukazawa J. Sakai T. Ishida S. Yamaguchi I. Kamiya Y. Takahashi Y. (2000). REPRESSION OF SHOOT GROWTH, a bZIP transcriptional activator, regulates cell elongation by controlling the level of gibberellins. Plant Cell. 12, 901–915. doi: 10.1105/tpc.12.6.901 10852936 PMC149092

[B15] Garg A. Kirchler T. Fillinger S. Wanke F. Stadelhofer B. Stahl M.. (2019). Targeted manipulation of bZIP53 DNA-binding properties influences arabidopsis metabolism and growth. J. Exp. Bot. 70, 5659–5671. doi: 10.1093/jxb/erz309 31257431 PMC6812703

[B16] Gomez C. Mitchell C. A. (2014). Growth responses of greenhouse tomato seedlings to different spectra of supplemental lighting are season-specific in a northern climate. HortScience 49, S273–S273.

[B17] Goujon T. Ferret V. Mila I. Pollet B. Ruel K. Burlat V.. (2003). Down-regulation of the *AtCCR1* gene in *Arabidopsis thaliana*: Effects on phenotype, lignins and cell wall degradability. Planta 217, 218–228. doi: 10.1007/s00425-003-0987-6 12783329

[B18] Gupta A. Hua L. Lin G. F. Molnár I. Doležel J. Liu S. Z.. (2021). Multiple origins of Indian dwarf wheat by mutations targeting the TREE domain of a GSK3-like kinase for drought tolerance, phosphate uptake, and grain quality. Theor. Appl. Genet. 134, 633–645. doi: 10.1007/s00122-020-03719-5 33164159

[B19] Hossain M. A. Cho J. I. Han M. Ahn C. H. Jeon J. S. An G.. (2010). The ABRE-binding bZIP transcription factor OsABF2 is a positive regulator of abiotic stress and ABA signaling in rice. J. Plant Physiol. 167, 1512–1520. doi: 10.1016/j.jplph.2010.05.008 20576316

[B20] Huang J. L. Gu M. Lai Z. B. Fan B. F. Shi K. Zhou Y. H.. (2010). Functional analysis of the arabidopsis *PAL* gene family in plant growth, development, and response to environmental stress. Plant Physiol. 153, 1526–1538. doi: 10.1104/pp.110.157370 20566705 PMC2923909

[B21] Jones L. Ennos A. R. Turner S. R. (2001). Cloning and characterization of *irregular xylem4* (*irx4*): a severely lignin-deficient mutant of *Arabidopsis* . Plant J. 26, 205–216. doi: 10.1046/j.1365-313x.2001.01021.x 11389761

[B22] Kato F. Araki M. Miyazawa Y. Fujii N. Takeda K. Suge H.. (2011). Factors responsible for deep-sowing tolerance in wheat seedlings: Varietal differences in cell proliferation and the coordinated synchronization of epidermal cell expansion and cortical cell division for the gibberellin-mediated elongation of first internodes. Ann. Bot. 108, 439–447. doi: 10.1093/aob/mcr173 21791455 PMC3158689

[B23] Khalaki M. A. Moameri M. Lajayer B. A. Astatkie T. (2021). Influence of nano-priming on seed germination and plant growth of forage and medicinal plants. Plant Growth Regulation 93, 13–28. doi: 10.1007/s10725-020-00670-9

[B24] Lee K. Back K. (2018). Melatonin-deficient rice plants show a common semidwarf phenotype either dependent or independent of brassinosteroid biosynthesis. J. Pineal Res. 66, e12537. doi: 10.1111/jpi.12537 30403303

[B25] Lee S. C. Choi H. W. Hwang I. S. Choi D. S. Hwang B. K. (2006). Functional roles of the pepper pathogen-induced bZIP transcription factor, CAbZIP1, in enhanced resistance to pathogen infection and environmental stresses. Planta 224, 1209–1225. doi: 10.1007/s00425-006-0302-4 16718483

[B26] Li X. Bonawitz N. D. Weng J. K. Chapple C. (2010). The growth reduction associated with repressed lignin biosynthesis in *Arabidopsis thaliana* is independent of flavonoids. Plant Cell. 22, 1620–1632. doi: 10.1105/tpc.110.074161 20511296 PMC2899864

[B27] Lin J. S. Huang X. X. Li Q. Cao Y. P. Bao Y. Meng X. F.. (2016). UDP-Glycosyltransferase 72B1 catalyzes the glucose conjugation of monolignols and is essential for the normal cell wall lignification in *Arabidopsis thaliana* . Plant J. 88, 26–42. doi: 10.1111/tpj.13229 27273756

[B28] Li J. Terzaghi W. Gong Y. Y. Li C. R. Ling J. J. Fan Y. Y.. (2020). Modulation of BIN2 kinase activity by HY5 controls hypocotyl elongation in the light. Nat. Commun. 11, 1592. doi: 10.1038/s41467-020-15394-7 32221308 PMC7101348

[B29] Liu Y. Fang Y. Huang M. J. Jin Y. L. Sun J. L. Tao X.. (2015). Uniconazole-induced starch accumulation in the bioenergy crop duckweed (*Landoltia punctata*) I: transcriptome analysis of the effects of uniconazole on chlorophyll and endogenous hormone biosynthesis. Biotechnol. Biofuels 8, 57. doi: 10.1186/s13068-015-0246-7 25866562 PMC4392464

[B30] Li Z. F. Zhang L. X. Yu Y. W. Quan R. D. Zhang Z. J. Zhang H. W.. (2011). The ethylene response factor AtERF11 that is transcriptionally modulated by the bZIP transcription factor HY5 is a crucial repressor for ethylene biosynthesis in arabidopsis. Plant J. 68, 88–99. doi: 10.1111/j.1365-313X.2011.04670.x 21645149

[B31] Lozano-Sotomayor P. Chávez Montes R. A. Silvestre-Vañó M. Herrera-Ubaldo H. Greco R. Pablo-Villa J.. (2016). Altered expression of the bZIP transcription factor DRINK ME affects growth and reproductive development in *Arabidopsis thaliana* . Plant J. 88, 437–451. doi: 10.1111/tpj.13264 27402171

[B32] Lv R. J. Zhang W. J. Xie X. B. Wang Q. J. Gao K. G. Zeng Y. H.. (2022). Foliar application uniconazole enhanced lodging resistance of high-quality indica rice (*Oryza sativa* l.) by altering anatomical traits, cell structure and endogenous hormones. Field Crops Res. 277, 108425. doi: 10.1016/j.fcr.2021.108425

[B33] Ma H. Z. Liu C. Li Z. X. Ran Q. J. Xie G. N. Wang B. M.. (2018). ZmbZIP4 contributes to stress resistance in maize by regulating ABA synthesis and root development. Plant Physiol. 178, 753–770. doi: 10.1104/pp.18.00436 30126870 PMC6181033

[B34] Oh E. Zhu J. Y. Bai M. Y. Arenhart R. A. Sun Y. Wang Z. Y. (2014). Cell elongation is regulated through a central circuit of interacting transcription factors in the arabidopsis hypocotyl. ELife 3, e03031. doi: 10.7554/eLife.03031 24867218 PMC4075450

[B35] Olsen K. M. Lea U. S. Slimestad R. Verheul M. and Lillo C. (2008). Differential expression of four *Arabidopsis PAL* genes; *PAL1* and *PAL2* have functional specialization in abiotic environmental-triggered flavonoid synthesis. J. Plant Physiol. 165, 1491–1499. doi: 10.1016/j.jplph.2007.11.005 18242769

[B36] Oyama T. Shimura Y. Okada K. (1997). The arabidopsis *HY5* gene encodes a bZIP protein that regulates stimulus-induced development of root and hypocotyl. Genes Dev. 11, 2983–2995. doi: 10.1101/gad.11.22.2983 9367981 PMC316701

[B37] Rohde A. Morreel K. Ralph J. Goeminne G. Hostyn V. De Rycke R.. (2004). Molecular phenotyping of the *pal1* and *pal2* mutants of *Arabidopsis thaliana* reveals far-reaching consequences on phenylpropanoid, amino acid, and carbohydrate metabolism. Plant Cell. 16, 2749–2771. doi: 10.1105/tpc.104.023705 15377757 PMC520969

[B38] Schomburg F. M. Bizzell C. M. Lee D. J. Zeevaart J. A. D. Amasino R. M. (2003). Overexpression of a novel class of gibberellin 2-oxidases decreases gibberellin levels and creates dwarf plants. Plant Cell. 15, 151–163. doi: 10.1105/tpc.005975 12509528 PMC143488

[B39] Shan C. Mei Z. L. Duan J. L. Chen H. Y. Feng H. F. Cai W. M. (2014). OsGA2ox5, a gibberellin metabolism enzyme, is involved in plant growth, the root gravity response and salt stress. PloS One 9, e87110. doi: 10.1371/journal.pone.0087110 24475234 PMC3903634

[B40] Shu K. Chen Q. Wu Y. R. Liu R. J. Zhang H. W. Wang S. F.. (2016). ABSCISIC ACID-INSENSITIVE 4 negatively regulates flowering through directly promoting arabidopsis FLOWERING LOCUS c transcription. J. Exp. Bot. 67, 195–205. doi: 10.1093/jxb/erv459 26507894 PMC4682436

[B41] Shu K. Luo X. F. Meng Y. J. Yang W. Y. (2018). Toward a molecular understanding of abscisic acid actions in floral transition. Plant Cell Physiol. 59, 215–221. doi: 10.1093/pcp/pcy007 29361058

[B42] Skubacz A. Daszkowska-Golec A. Szarejko I. (2016). The role and regulation of ABI5 (ABA-insensitive 5) in plant development, abiotic stress responses and phytohormone crosstalk. Front. Plant Sci. 7, 1884. doi: 10.3389/fpls.2016.01884 28018412 PMC5159420

[B43] Song S. Y. Liu G. Z. Ma F. F. Bao Z. L. (2022). Brassinazole represses tomato hypocotyl elongation *via* inhibition of cell division. Plant Growth Regulation 96, 463–472. doi: 10.1007/s10725-022-00798-w

[B44] Tak H. Mhatre M. (2013). Cloning and molecular characterization of a putative *bZIP* transcription factor *VvbZIP23* from *Vitis vinifera* . Protoplasma 250, 333–345. doi: 10.1007/s00709-012-0417-3 22610648

[B45] Tao L. X. Wang X. Yu M. Y. Huang X. L. Xu R. S. (1997). Effects of environmental conditions on degradation of s-07 (uniconazole, pentefezole) and PP333 (MET, paclobutrazol) in soil. Acta Agricult. Zhejiangensis 9, 246–250.

[B46] Tesfahun W. Menzir A. (2018). Effect of rates and time of paclobutrazol application on growth, lodging, yield and yield components of tef [*Eragrostis tef* (Zucc.) trotter] in ada woreda, East shewa, Ethiopia. J. Biol. Agric. Healthc. 8, 104–117.

[B47] Van Leene J. Blomme J. Kulkarni S. R. Cannoot B. De Winne N. Eeckhout D.. (2016). Functional characterization of the arabidopsis transcription factor bZIP29 reveals its role in leaf and root development. J. Exp. Bot. 67, 5825–5840. doi: 10.1093/jxb/erw347 27660483 PMC5066499

[B48] Vernoux T. Brunoud G. Farcot E. Morin V. Van den Daele H. Legrand J.. (2011). The auxin signalling network translates dynamic input into robust patterning at the shoot apex. Mol. Syst. Biol. 7, 508. doi: 10.1038/msb.2011.39 21734647 PMC3167386

[B49] Wang Y. P. Kang Y. Y. Zhong M. Zhang L. Chai X. R. Jiang X. X.. (2022). Effects of iron deficiency stress on plant growth and quality in flowering Chinese cabbage and its adaptive response. Agronomy 12, 875. doi: 10.3390/agronomy12040875

[B50] Weiste C. Pedrotti L. Selvanayagam J. Muralidhara P. Fröschel C. Novák O.. (2017). The arabidopsis bZIP11 transcription factor links low-energy signalling to auxin-mediated control of primary root growth. PloS Genet. 132, e1006607. doi: 10.1371/journal.pgen.1006607 PMC5315408 28158182

[B51] Yan J. D. Liao X. Y. He R. Q. Zhong M. Feng P. P. Li X. M.. (2017). Ectopic expression of *GA 2-oxidase 6* from rapeseed (*Brassica napus* l.) causes dwarfism, late flowering and enhanced chlorophyll accumulation in *Arabidopsis thaliana* . Plant Physiol. Biochem. 111, 10–19. doi: 10.1016/j.plaphy.2016.11.008 27886559

[B52] Yan J. D. Xiang F. J. Yang P. Li X. Zhong M. He R.. (2021). Overexpression of *BnGA2ox2*, a rapeseed gibberellin 2-oxidase, causes dwarfism and increased chlorophyll and anthocyanin accumulation in arabidopsis and rapeseed. Plant Growth Regulation 93, 65–77. doi: 10.1007/s10725-020-00665-6

[B53] Yue L. Q. Li Y. S. Zhong M. Chai X. R. Zhao P. Y. Huang R. M.. (2022). Benzoic acid, chlorine dioxide, and 1-methylcyclopropene induce flavonoid metabolic shifts in postharvest flowering Chinese cabbage revealed by high-dimensional analytical data. Int. J. Mol. Sci. 23, 6011. doi: 10.3390/ijms23116011 35682691 PMC9180784

[B54] Zhang L. L. Li Z. P. Garraway J. Cai Q. Z. Zhou Y. F. Li X.. (2020). The casein kinase 2 β subunit CK2B1 is required for swollen stem formation *via* cell cycle control in vegetable *Brassica juncea* . Plant J. 104, 706–717. doi: 10.1111/tpj.14958 32772441

[B55] Zhang W. F. Tan L. B. Sun H. Y. Zhao X. H. Liu F. X. Cai H. W.. (2019). Natural variations at *TIG1* encoding a TCP transcription factor contribute to plant architecture domestication in rice. Mol. Plant 12, 1075–1089. doi: 10.1016/j.molp.2019.04.005 31002981

[B56] Zhao Q. Nakashima J. Chen F. Yin Y. B. Fu C. X. Yun J. F.. (2013). *LACCASE* is necessary and nonredundant with *PEROXIDASE* for lignin polymerization during vascular development in arabidopsis. Plant Cell. 25, 3976–3987. doi: 10.1105/tpc.113.117770 24143805 PMC3877815

[B57] Zhou H. Liang X. Y. Feng N. J. Zheng D. F. Qi D. Q. (2021). Effect of uniconazole to soybean seed priming treatment under drought stress at VC stage. Ecotoxicol. Environ. Safety 224, 112619. doi: 10.1016/j.ecoenv.2021.112619 34403945

[B58] Zhou T. M. Wu Z. Wang Y. C. Su X. J. Qin C. X. Huo H. Q.. (2019). Modelling seedling development using thermal effectiveness and photosynthetically active radiation. J. Integr. Agricult. 18, 2521–2533. doi: 10.1016/S2095-3119(19)62671-7

[B59] Zuo Q. S. Wang L. Zheng J. D. You J. J. Yang G. Leng S. H.. (2020). Effects of uniconazole rate on agronomic traits and physiological indexes of rapeseed blanket seedling. Oil Crop Sci. 5, 198–204. doi: 10.1016/j.ocsci.2020.12.003

