# Supplementary material for: Transcriptomic and metabolomic analyses reveal the mechanism of uniconazole inducing hypocotyl dwarfing by suppressing BrbZIP39–BrPAL4 module mediating lignin biosynthesis in flowering Chinese cabbage
Source: Front Plant Sci. 2022 Dec 14;13:1014396. doi: 10.3389/fpls.2022.1014396 (PMC9794620; doi:10.3389/fpls.2022.1014396)
Supplement: Supplementary file 1 [file DataSheet_1.pdf]

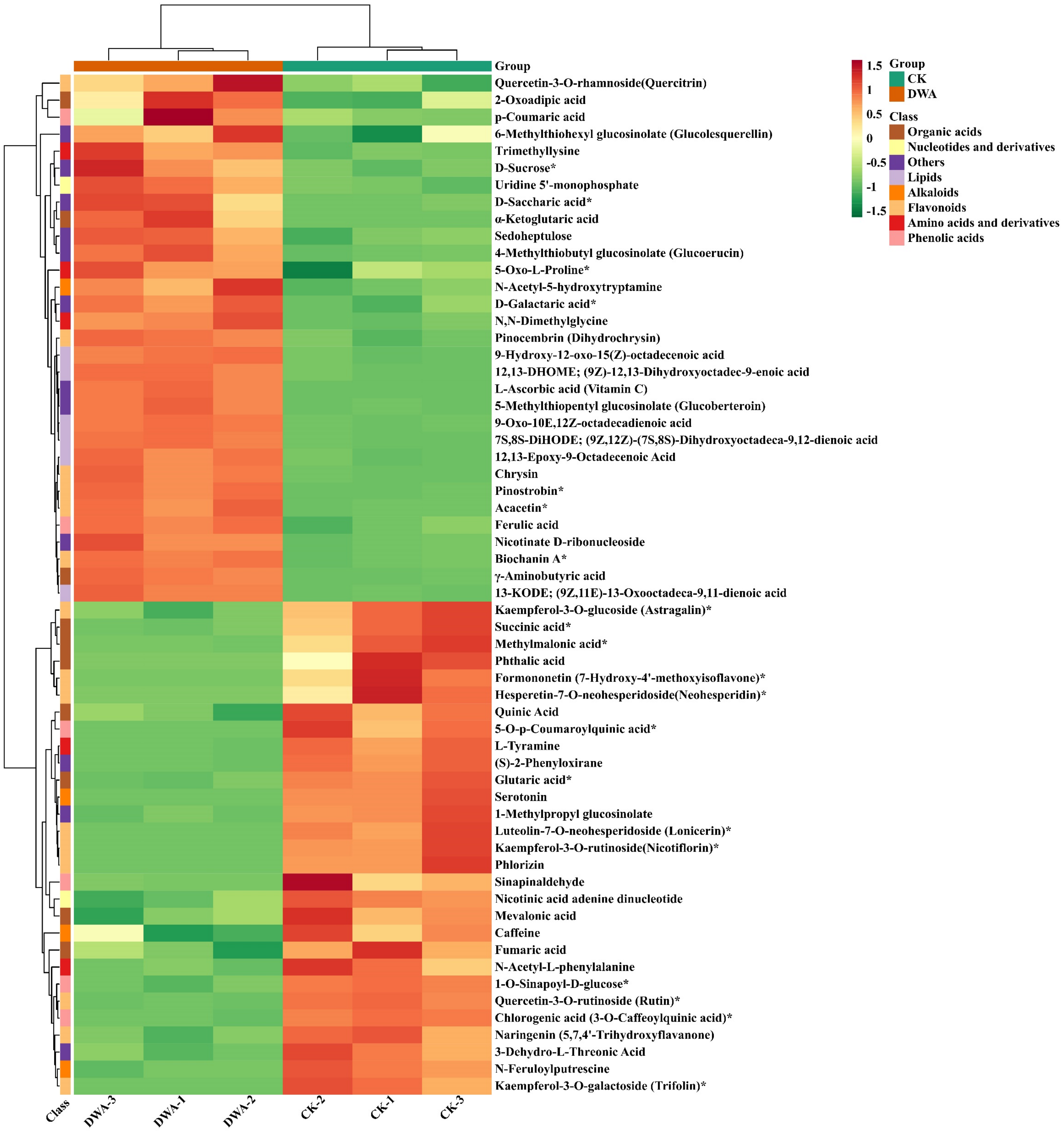

Figure S1. The differential metabolite clustering heatmap of KEGG pathway.

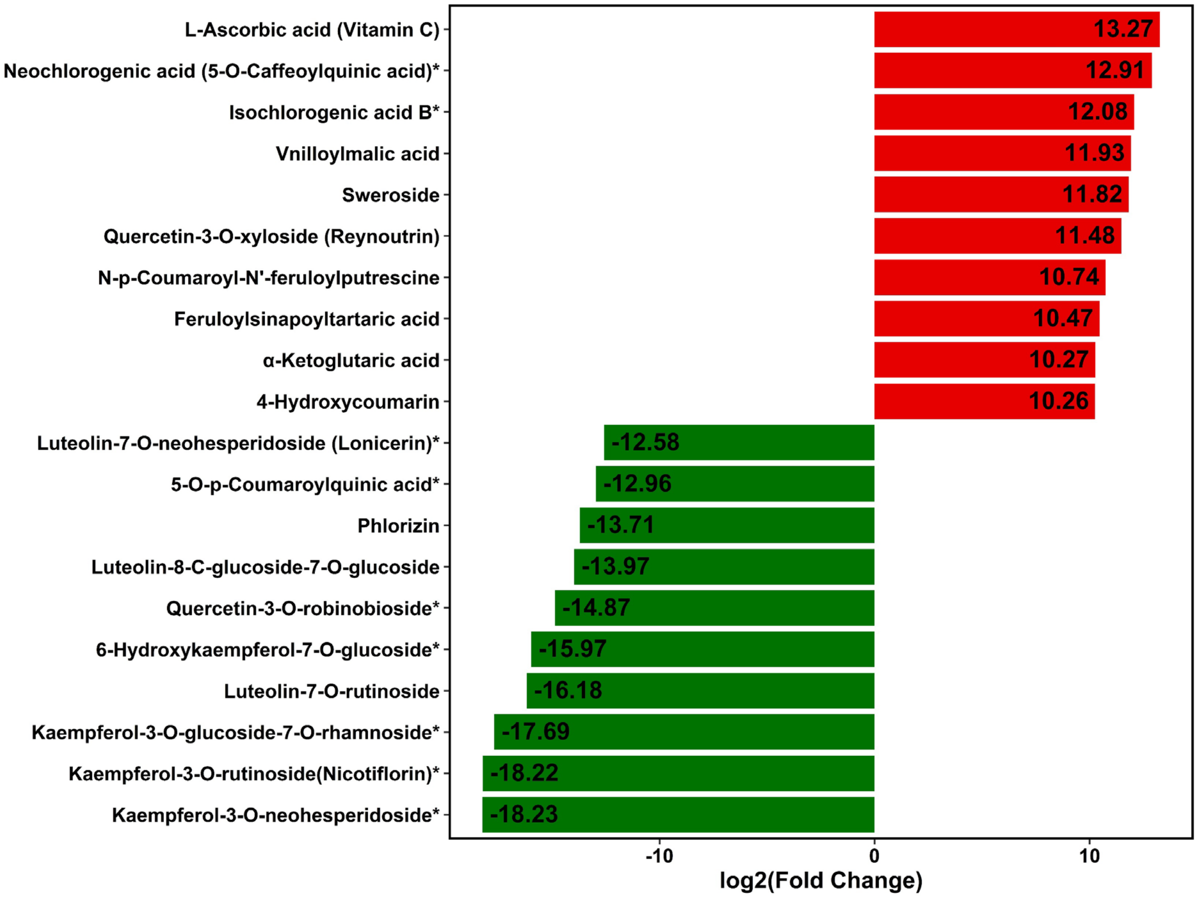

Figure S2. The bar chart of top 10 upregulating and downregulating metabolites

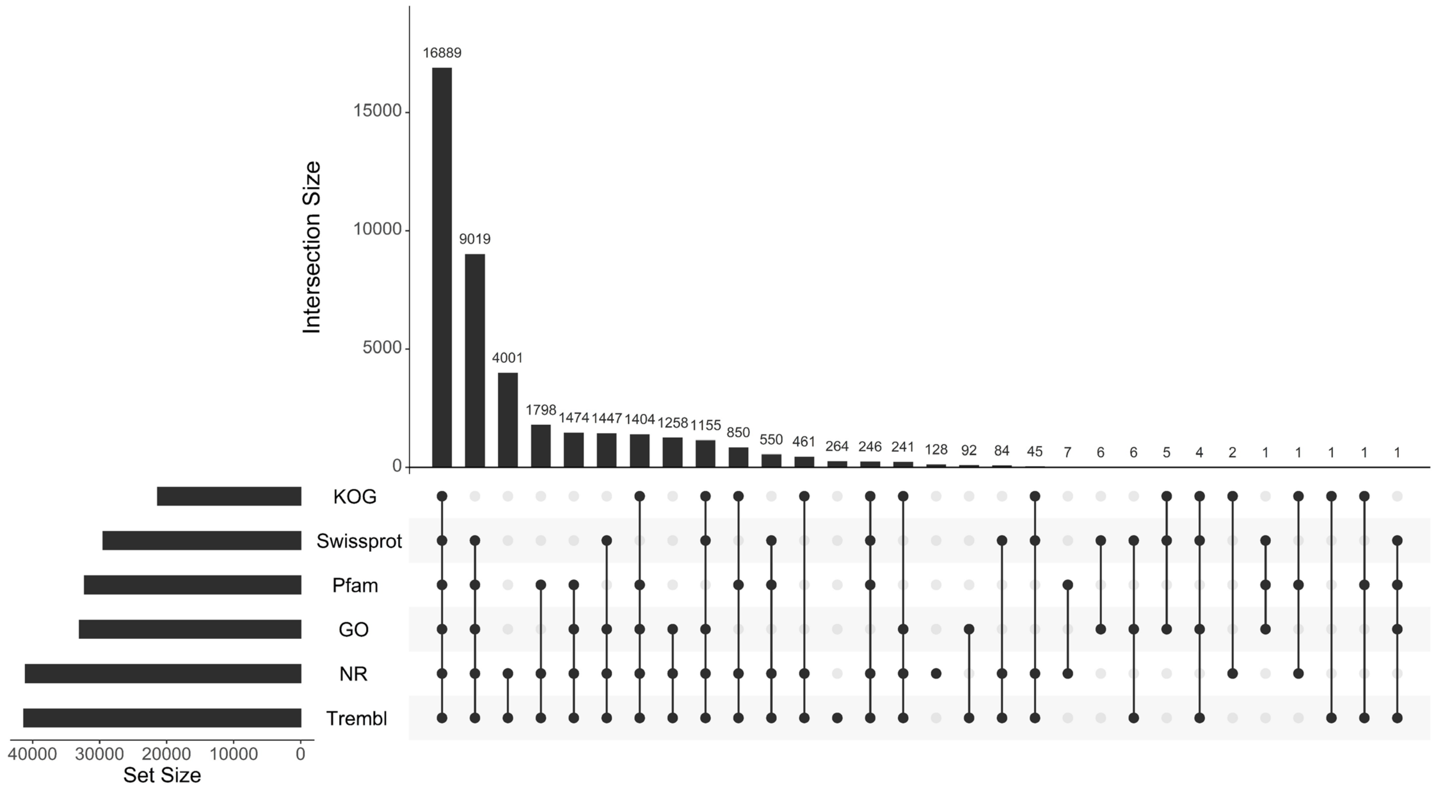

Figure S3. Transcriptome functional annotation. All transcripts were annotated through homology based sequence comparison across six database, i.e., eukaryotic orthologous groups (KOG), (Swissprot), Swissprot, Pfam, Gene Ontology (GO), NCBI non-redundant protein sequences (NR), Trembl.

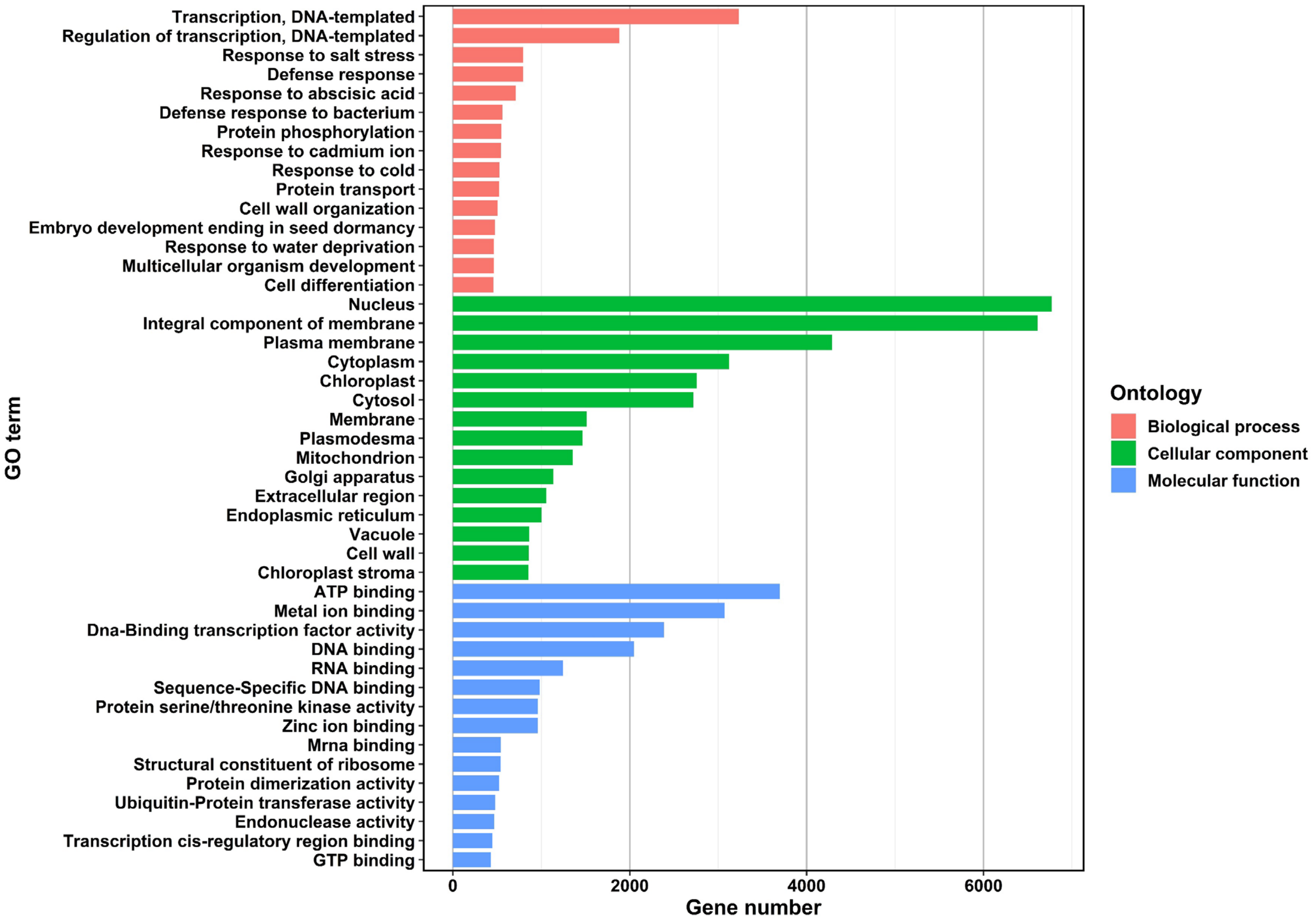

Figure S4. GO terms of transcripts in transcriptome data.

A

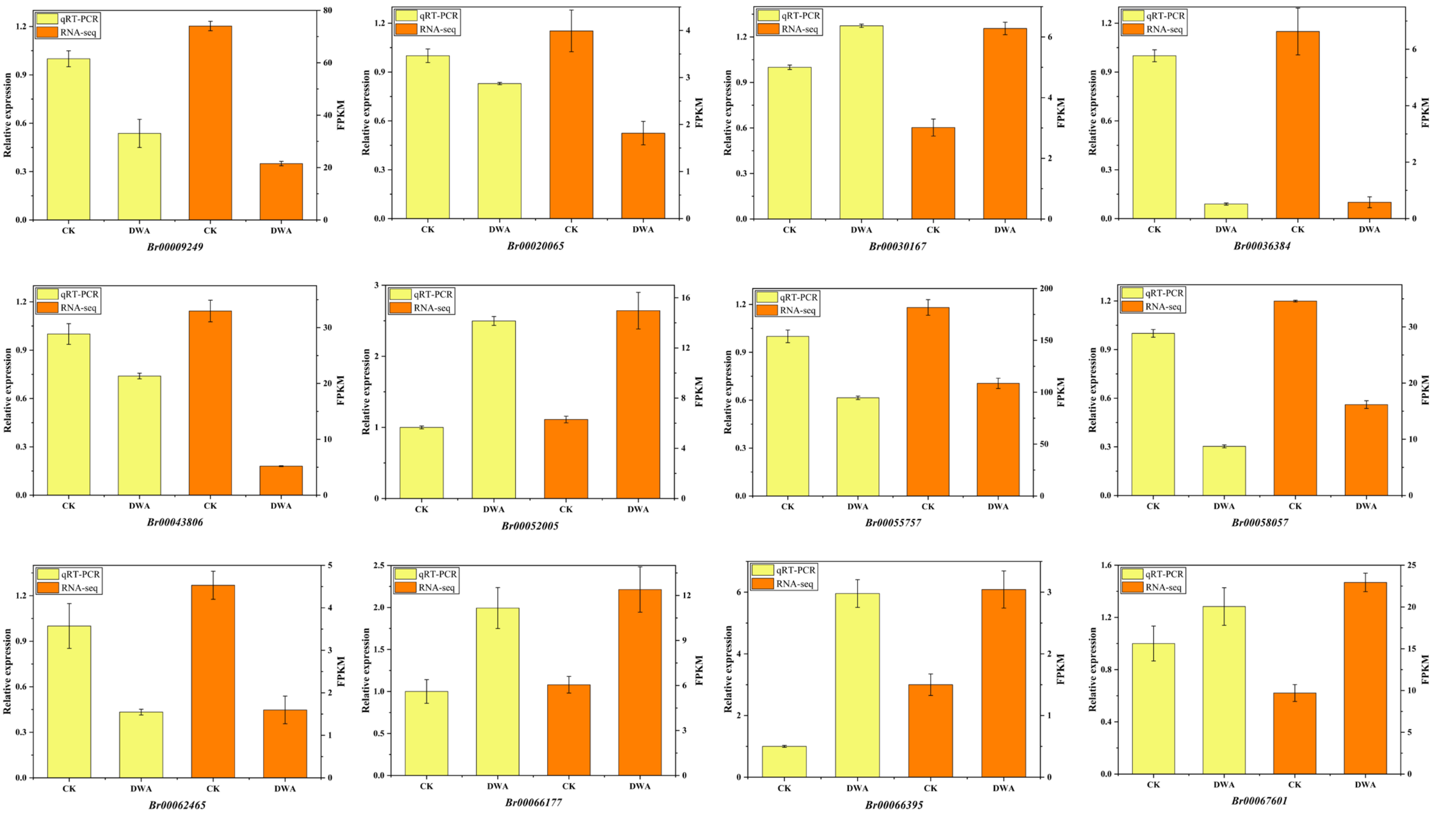

B

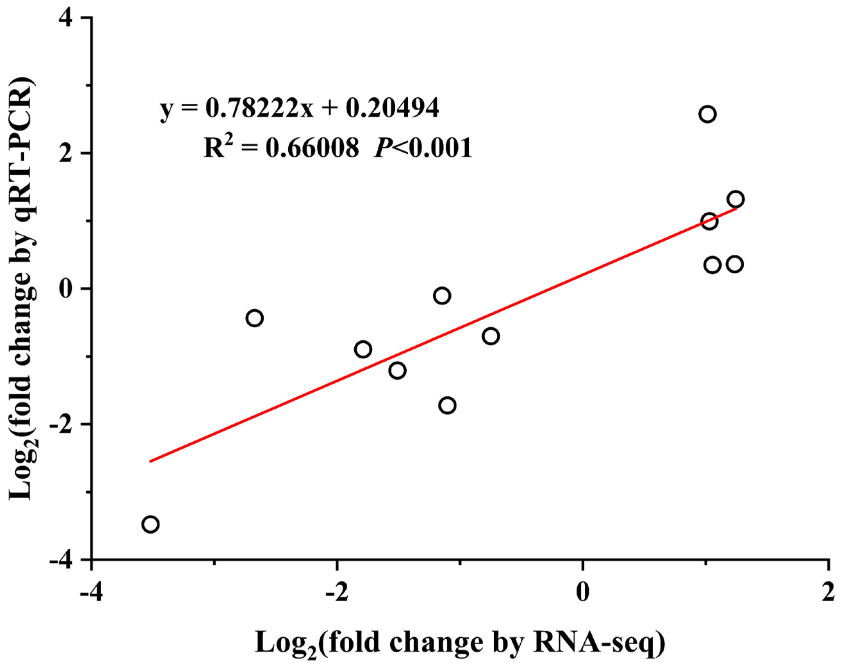

Figure S5. Correlation between the results from RNA-seq and qRT-PCR. **(A)** Relative expression and FPKM of 12 randomly selected DEGs. **(B)** Correlation between the results from RNA-seq and qRT-PCR of 12 randomly selected DEGs.

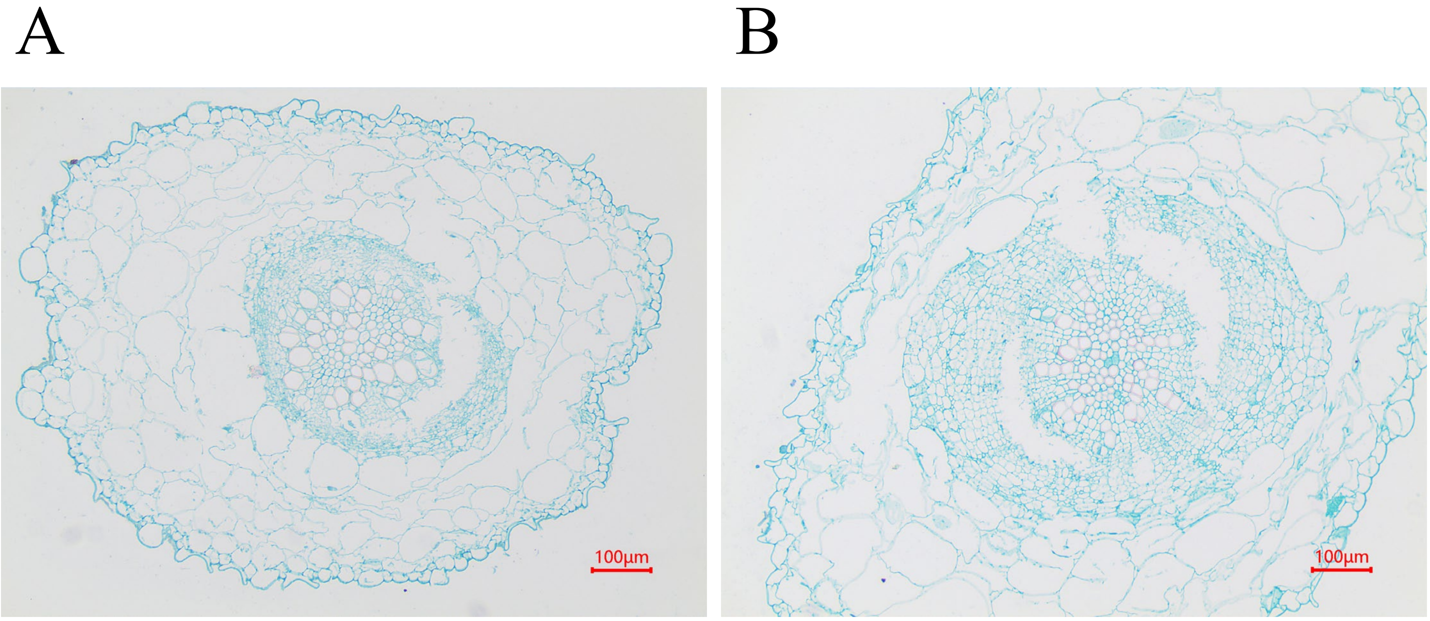

Figure S6. The cross sections in hypocotyl of flowering Chinese cabbage seedlings under CK**(A)** and DWA **(B)**.

Supplementary Table S1. RNA-Seq data information.

Sample	Raw Reads	Clean Reads	Clean base (G)	Error Rate (%)	Q20 (%)	Q30 (%)	GC (%)
CK-1	45157056	42991654	6.45	0.03	97.71	93.4	46.66
CK-2	52328830	49681420	7.45	0.03	97.79	93.75	46.73
CK-3	48192930	45847828	6.88	0.03	97.93	93.94	46.66
DWA-1	49153450	46976462	7.05	0.03	97.73	93.57	46.79
DWA-2	47404826	45269550	6.79	0.03	97.83	93.81	46.8
DWA-3	47761584	45721814	6.86	0.03	97.77	93.64	46.59

Supplementary Table 2. The major *cis*-acting elements of *BrPAL4* promoter.

***cis*-acting element**	**Sequence**	**Function**	**Number**
TATA-box	TATAAA/TATAA/TATA/TACATAAA/TATATA	core promoter element around -30 of transcription start	11
ABRE	ACGTG/CACGTG	cis-acting element involved in the abscisic acid responsiveness	9
CAAT-box	CCAAT/TGCCAAC/CAAAT	common cis-acting element in promoter and enhancer regions	7
G-box	TACGTG/CACGTG/CACGTC	cis-acting regulatory element involved in light responsiveness	5
G-Box	CACGTT/CACGTG	cis-acting regulatory element involved in light responsiveness	4
GT1-motif	GGTTAA	light responsive element	2
AE-box	AGAAACTT	part of a module for light response	2
CGTCA-motif	CGTCA	cis-acting regulatory element involved in the MeJA-responsiveness	2
TGACG-motif	TGACG	cis-acting regulatory element involved in the MeJA-responsiveness	2
I-box	gGATAAGGTG/AGATAAGG	part of a light responsive element	2
TCT-motif	TCTTAC	part of a light responsive element	1
GCN4_motif	TGAGTCA	cis-regulatory element involved in endosperm expression	1
GARE-motif	TCTGTTG	gibberellin-responsive element	1
TCA-element	TCAGAAGAGG	cis-acting element involved in salicylic acid responsiveness	1

Supplementary Table S3. The primers used for qRT-PCR.

Primer Name	Primer Sequence (5' to 3')
GAPDH-F	CCGCTAACTGCCTTGCTCCACTT
GAPDH-R	GCGGCTCTTCCACCTCTCCAGT
Br00009249-F	TACGCGGACGATCCTTGC
Br00009249-R	TCAGCTCCGCCTCAAACG
Br00020065-F	AAGCGAGGGGGCATAGGA
Br00020065-R	CCAAGTCCAGCGGCAAGA
Br00030167-F	AAGCCGCCGCATAGGTAC
Br00030167-R	TTGCGGAAGACACGGACC
Br00036384-F	CGACACAGAGTCTGCGAGT
Br00036384-R	GTGTCCAGCCAAGCGTCT
Br00043806-F	CACCACAATCTCCGGCGT
Br00043806-R	GCGCCGGTGATGTTGTTG
Br00052005-F	GGGATCGTGACGTGGCAA
Br00052005-R	GGCTTGAAAGGCGGGTGA

Supplementary Table S3. The primers used for qRT-PCR.

Primer Name	Primer Sequence (5' to 3')
Br00055757-F	TGAACTGGGGTGCAGCAG
Br00055757-R	GCCTACGGTGGAGATCGC
Br00058057-F	TGTCGTGGCGGAAGGTTC
Br00058057-R	CGTGCTCTTCCTCCGTCC
Br00062465-F	CTGATGCCGCAGTTTGCC
Br00062465-R	CATCCCGTGGCCTGAGTC
Br00066177-F	CAGGTCAGCAGATCCCGC
Br00066177-R	CACCGGCTGTAGCGTCTC
Br00066395-F	AGAGCGGGAATGCAGCAG
Br00066395-R	TCCGGTTGTACTGCTCCG
Br00067601-F	CTGACCGAGTCCCCTGGA
Br00067601-R	CAGCTTCTCCACCTCGCC
